# Surface-Enhanced Resonance Raman Scattering of Rhodamine 6G in Dispersions and on Films of Confeito-Like Au Nanoparticles

**DOI:** 10.3390/s17112563

**Published:** 2017-11-07

**Authors:** Masaki Ujihara, Nhut Minh Dang, Toyoko Imae

**Affiliations:** 1 Graduate Institute of Applied Science and Technology, National Taiwan University of Science and Technology, 43 Keelung Road, Section 4, Taipei 10607, Taiwan; imae@mail.ntust.edu.tw; 2 Department of Chemical Engineering, National Taiwan University of Science and Technology, 43 Keelung Road, Section 4, Taipei 10607, Taiwan; dangmnhut@gmail.com

**Keywords:** surface enhanced Raman scattering, surface enhanced resonance Raman scattering, gold nanoparticle, non-spherical nanoparticle, localized surface plasmon

## Abstract

Surface-enhanced resonance Raman scattering (SERRS) of rhodamine 6G was measured on confeito-like Au nanoparticles (CAuNPs). The large CAuNPs (100 nm in diameter) in aqueous dispersion systems showed stronger enhancing effect (analytical enhancement factor: over 10^5^) of SERRS than the small CAuNPs (50 nm in diameter), while the spherical Au nanoparticles (20 nm in diameter) displayed rather weak intensities. Especially, minor bands in 1400–1600 cm^−1^ were uniquely enhanced by the resonance effect of CAuNPs. The enhancement factors revealed a concentration dependence of the enhancing effect at low concentration of rhodamine 6G. This dependency was due to a large capacity of hot-spots on CAuNPs, which were formed without agglomeration. The surface-enhancing behaviour in the film systems was similar to that in the dispersions, although the large CAuNPs had lower enhancing effect in the films, and the small CAuNPs and the spherical Au nanoparticles were more effective in their films. These results suggest that the CAuNPs have an advantage in ultrasensitive devices both in dispersions and films, compared to the agglomerate of spherical Au nanoparticles.

## 1. Introduction

Over the ages, nanoparticles of noble metals have been used as pigments [1]. They can efficiently absorb the light at a specific band, and their optical behavior is today understood as a resonance of collective motion of surface electrons with the incident light [1,2,3]. This phenomenon, named localized surface plasmon resonance (LSPR), depends on many factors like the size and morphology of the nanoparticles and the dielectric constant of the surrounding medium [1,2,3,4], and the changes in these factors cause the shift and intensification of plasmon absorption band of the nanoparticles. Thus, the LSPR phenomenon is used in sensing devices, as the stimulation changes the color of nanoparticles [5]. On the contrary, the motion of electrons on the surface can affect the other materials adjacent to the nanoparticle. The incident light polarizes the nanoparticle through the LSPR, and it provides the oscillating electric field around the nanoparticle [1,2,3,4,5,6]. Then, the materials on the nanoparticle can be strongly excited by this localized electric field. This phenomenon is useful to intensify the spectra of the adsorbate on the nanoparticles, and a spectroscopy using this enhancing effect is called the surface-enhanced spectroscopy [6,7]. Today, surface-enhanced Raman scattering (SERS) [8,9,10], infrared absorption [11,12,13,14,15,16,17,18,19,20,21,22], and fluorescence spectroscopies [23,24,25] are used for ultrasensitive analyses.

To improve these enhancing effects, the LSPR can be tuned by designing the size and morphology of nanoparticles. The localization of energy typically occurs at junctions and tips of nanoparticles, and such areas are called “hot-spots” [26,27,28]. Then, the nanoparticles have been developed to form the hot-spots: Agglomerates of nanoparticles were fabricated to provide the junctions [26,27,28,29], and non-spherical nanoparticles were designed to focus the energy at the tips on nanoparticles [30,31,32,33,34]. These nanoparticles can be assembled on metal substrates to improve the enhancing effect [32,33,34] or used as dispersions to measure the SERS in fluids [29]. While the agglomerates of conventional nanoparticles have difficulty in reproducibility, the non-spherical nanoparticles with well-defined structures have advantage to tune the properties of LSPR and can provide the hot-spots in dispersions without agglomeration [35,36,37]. Furthermore, the resonance effects between the surface plasmon and the molecular bands of target have been considered to intensify the SERS [38,39,40,41]. This effects were used for the surface-enhanced resonance Raman scattering (SERRS), and then the tuning in LSPR to the molecular bands was investigated [40,41].

In our study, the enhancing effects of non-spherical Au nanoparticles (AuNPs) were examined. The synthesis of confeito-like AuNPs (spherical AuNPs with many projections on their surfaces: CAuNPs) and their SERS effect have been reported in 2008 [42], and the synthesis method was improved in viewpoint of green chemistry and morphology control [43,44,45,46]. In the present study, the analytical enhancement factors of SERS were evaluated for the CAuNPs. The resonance effects were compared by using AuNPs with different sizes in dispersions and on films, and the capture ability of hot-spots was also discussed. This work will be useful to design the plasmonic devices for ultrahighly sensitive analyses.

## 2. Materials and Methods

### 2.1. Reagents

Sodium tetrachloroaurate(III) dihydrate (NaAuCl_4_∙2H_2_O, 99%) was purchased from Sigma Aldrich Co. (St. Louis, MO, USA). Anhydrous citric acid (Cit), sodium hydroxide (NaOH), Rhodamine 6G (R6G) and an aqueous solution of hydrogen peroxide (35 wt %, H_2_O_2_) were purchased from Acros Organics (Morris Plains, NJ, USA). All chemicals were of reagent grade and were used without further purification. Ultrapure water (Yamato Millipore WT100, Tokyo, Japan) with a resistivity of 18.2 MΩ·cm was used throughout all the syntheses and measurements in the study.

### 2.2. Synthesis of AuNPs

CAuNPs were synthesized using H_2_O_2_ as reducing agent as previously reported [21,22,23]. An aqueous solution of NaAuCl_4_ (4 mL, 0.1 mM) was mixed with aqueous solutions of citric acid (28 mL, 15.6 mg or 31.2 mg), and then H_2_O_2_ (160 µL, 35 wt %) was added to the solution. Finally, 8 mL of an aqueous solution of NaOH (100 mM) was mixed with vigorous stirring for 1 min. The reaction solution was allowed to stand overnight to accomplish the reaction, and a dispersion of AuNPs was obtained. For the synthesis of spherical AuNPs, 4 mL of an aqueous solution of NaAuCl_4_ (1 mM) was diluted by 35 mL of water and refluxed with stirring. Then a solution of trisodium citrate (1 mL, 1 wt %) was added into the solution and kept boiling for 5 min until the color of the solution changed into red. The solution was continuously stirred for 1 min and then cooled down to the room temperature.

### 2.3. Instruments

Transmission electron microscopic (TEM) images were taken with a H-7000 instrument (Hitachi, Tokyo, Japan) at an accelerating voltage of 100 kV. The dispersion of AuNPs was dropped on a carbon-coated copper grid, air-dried, and then used for the observation. Ultraviolet-visible-near infrared (UV-vis-NIR) absorption spectra were recorded with a V-670 spectrophotometer (JASCO, Tokyo, Japan) with quartz cells of 1 and 10 mm light path for the dispersion. Raman scattering was measured with an iHR550 imaging spectrometer (1800 gr/mm grating and slit opening of 300 μm) from Horiba Jobin Yvon (Kyoto, Japan) equipped with a confocal microscope (object lens: MPlan N 50X, Olympus, Tokyo, Japan, working distance of 0.38 mm).

### 2.4. Measurement of SERS in Dispersions and on Films of AuNPs

The dispersions of AuNPs (originally 0.1 mM/Au) were concentrated to 5 mM/Au by centrifugation (10,000 min^−1^, 5590× *g*, 10 min). An aqueous solution of R6G (1 mM, 2–100 µL) was added to the dispersion of AuNPs (5 mM/Au, 200 µL). The mixture was diluted by water to 400 µL (2.5 mM/Au, 0.5–250 μM/R6G), and then, 20 µL of the mixture was dropped on a silicon substrate. The mixture was covered by a thin glass plate with a spacer of 1 mm height to prevent drying during the SERS measurement. For the film system, the dispersion of AuNP (5 mM/Au, 20 µL) was dropped inside a circle of 5 mm in diameter on a silicon substrate and dried. Subsequently, an aqueous solution of R6G (20 µL, 0.5–250 μM) was put on the film and covered by the glass plate for measurements. Both for dispersion systems and for film systems, the SERS measurement was performed with the excitation wavelength of 632.8 nm and the laser power of 10 mW. Using the microscope, the focusing was carried out on the AuNPs in dispersions beneath the cover glass. For the films, the laser focus was adjusted on the aggregates of AuNPs to acquire the maximum Raman intensity. Then, the Raman spectrum was recorded with 8 accumulations of 60 s exposure time for each spot. 

## 3. Results and Discussion

### 3.1. Characterization of AuNPs

**Figure 1:**
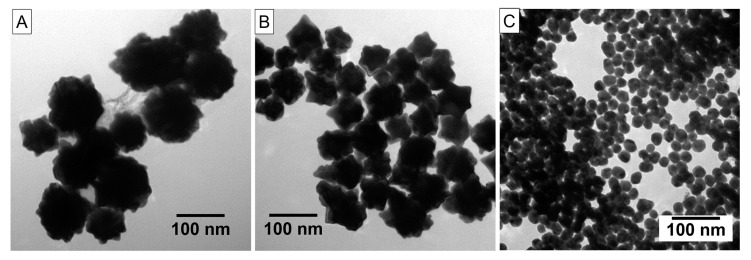
TEM images of AuNPs after concentration: (**A**) large CAuNPs, (**B**) small CAuNPs, and (**C**) spherical AuNPs.

**Figure 2:**
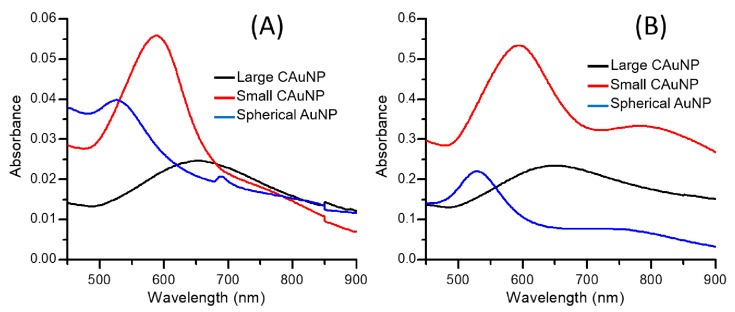
UV-Vis spectra of CAuNPs and spherical AuNPs (**A**) before and (**B**) after concentration. The light paths were 10 mm for A and 1 mm for B, respectively. The concentrations were 0.1 mM/Au before centrifugation and 5 mM/Au after centrifugation.

The CAuNPs of two different sizes and the conventional spherical AuNPs were prepared as previously reported [43,44,45,46]. The average sizes of CAuNPs measured from TEM images were 100 and 50 nm (Figure 1A,B) for the preparation at low and high concentrations of citric acid, respectively. The spherical AuNPs were 20 nm in diameter as conventionally obtained [47]. These AuNPs in dispersions had their specific plasmon absorption bands at 655 nm (100 nm CAuNPs), 590 nm (50 nm CAuNPs), and 530 nm (spherical AuNPs) (Figure 2). The red-shifted plasmon absorption bands of the CAuNPs in dispersions suggest a convergence of the surface plasmon on the tips, which is caused by intra-particle interactions of surface plasmon on the surface of individual particle [26,43,44,45,46]. When the dispersions were concentrated to 5 mM/Au, the 50 nm CAuNPs showed a new broad absorption band around 800 nm in addition to the original plasmon adsorption band, although the 100 nm CAuNPs had the absorption band at the same position (Figure 2). Similarly, the spherical AuNPs indicated a new broad absorption band around 730 nm with the original band. These new bands suggest that the small AuNPs (50 nm confeito-like and 20 nm spherical, both) agglomerated by the centrifugation (Figure 1B,C), following the shift of their plasmon absorption bands by the inter-particle interactions [5,26].

### 3.2. SERS of Rhodamine 6G in Dispersions of AuNPs

**Figure 3:**
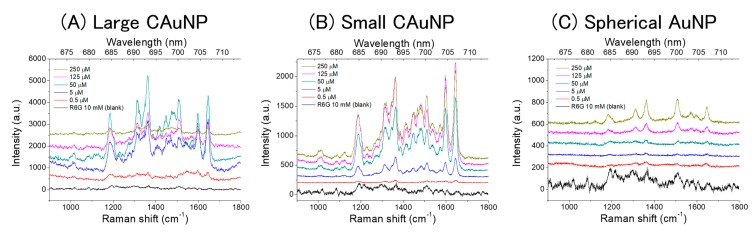
Raman scattering of R6G in the dispersions of CAuNPs and spherical AuNPs. (**A**) large CAuNP, (**B**) small CAuNP, and (**C**) spherical AuNP.

The Raman scattering of R6G was measured to evaluate the surface-enhancing effects of the AuNPs. The measurements were performed in two different conditions; in dispersions and on films of AuNPs. The obtained Raman spectra in the dispersions of AuNPs were shown in Figure 3 with the spectrum of a control solution of R6G (10 mM, without AuNPs). The negative control (without R6G) didn’t provide significant signals (Figure S1 in Supplementary Materials). Observed strong Raman bands, especially at 1183, 1363, 1600, and 1645 cm^−1^, differed in position from those of corresponding bands of the control solution (1192, 1366, 1606, and 1649 cm^−1^, respectively) and thus the strong surface-enhancing effects of the AuNPs were demonstrated [38,39] The spherical AuNPs indicated the significantly weaker SERS than the CAuNPs, as expected [43,46]. However, the spectral profiles on the CAuNPs were different from those on the spherical AuNPs: small bands in the range of 1400–1600 cm^−1^ were characteristic for the SERS from CAuNPs and identified as the bands at 1412, 1449, 1465, 1482, 1508, and 1570 cm^−1^ (see Figure S2 in Supplementary Materials), while these were very weak in the SERS on the spherical AuNPs. This difference indicates that the CAuNPs provided a particularly strong enhancement in this region, while the spherical AuNPs had less resonance effect there [38,39,40,41]. The difference between their resonance effects was consistent with the different ratio of spectral intensities between bands at 1508 and 1645 cm^−1^; the CAuNPs enhanced the latter more than the former (Figure 3A,B), as seen in the typical SERRS [38,39]. However, the intensities of bands in the range of 1400–1500 cm^−1^ were similar to that of the band at 1508 cm^−1^, although they were very weak in the typical SERRS [38,39] and even in the SERRS with the CAuNPs using the excitation wavelength at 532 and 785 nm in our previous studies [43,46]. This implies a unique mechanism for the SERRS using the CAuNPs.

On the other hand, a broad emission was also observed in the range from 1200 to 1700 cm^−1^ where the characteristic bands in the SERRS from CAuNPs appeared (Figure 3A,B). This emission could be the the surface-enhanced fluorescence of R6G [23,24,25,40,41], which were not observed when the excitation wavelength was 532 and 785 nm in our previous studies because these wavelengths didn’t sufficiently match with the plasmon absorption bands of CAuNPs [43,46]. Therefore, the unique enhancement in the range of 1400–1500 cm^−1^ in the present study could be explained by the two-fold electromagnetic enhancement theory derived by Ozaki et al. [41]: The two-step LSPR of AuNPs with both the excitation light and the emission (of Raman-scattering light and fluorescence from R6G) enhances the Raman scattering and the fluorescence. In this mechanism, the SERRS is extraordinarily intensified in the range where the plasmon resonance Rayleigh scattering and the surface-enhanced fluorescence appear. For the CAuNPs, the proximity of the excitation wavelength (632.8 nm), surface plasmon bands of CAuNPs (655 nm and 590 nm, Figure 2) and the molecular band of R6G adsorbed on AuNP (590–620 nm as *J*-type aggregates) allowed the two-fold mechanism [41,48]. The absorption band of single R6G (~530 nm) didn’t contribute to this strong SERRS mechanism [46]. This mechanism didn’t effectively work for the spherical AuNPs to intensify the bands in the range (1400–1600 cm^−1^) (Figure 3C), because the emission from the aggregates of R6G had lower energy than the LSPR of isolated AuNPs (530 nm) and too far from that of agglomerates (730 nm).

In the dispersion of large CAuNPs, the intensity of SERRS decreased at high concentration of R6G (Figure 3A). This decreasing can be explained by the agglomeration and the precipitation of AuNPs caused by the addition of excess R6G. The precipitation in the test tube was also observed by the naked eyes. Because AuNPs are stabilized by negatively charged –COO^−^ groups of citrate [43,45,47], the R6G molecules can strongly adsorb on the surface of AuNPs via the electrostatic interaction to neutralize R6G-adsorbed AuNPs and to cause the agglomeration of the particles. Generally, the large particle has the less specific surface area than the small particle possesses. Thus, the large CAuNPs suffered the precipitation easier than the other small AuNPs did. Therefore, the evaluation of SERS should be done at the low concentration to avoid the precipitation.

### 3.3. SERS of Rhodamine 6G on Films of AuNPs

**Figure 4:**
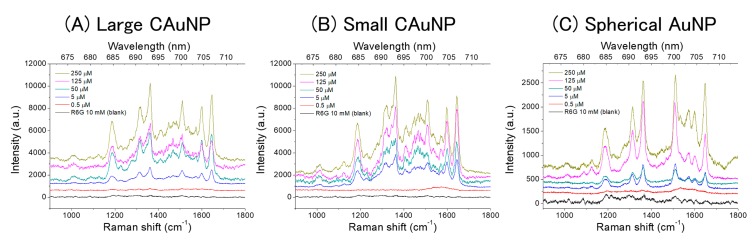
Raman scattering of R6G on the films of CAuNPs and spherical AuNPs. (**A**) large CAuNP, (**B**) small CAuNP, and (**C**) spherical AuNP.

The AuNPs were dried on the Si substrate and used for the SERS measurements. The AuNPs were considered to aggregate and form films (see Figure S3 in the Supplementary Materials) [45,46]. For the films of AuNPs, the obtained Raman spectra are shown in Figure 4.

Their intensities were mostly stronger than those from the corresponding dispersions, except the large CAuNPs at low concentration (Figure 3 and Figure 4). The intensities of SERS from the film systems of small CAuNPs resembled those of the corresponding large CAuNPs (Figure 4A,B), and their shapes were similar to those from the dispersion systems. These imply that the film formation of CAuNPs does not largely change their LSPR properties, and the obtained spectra are SERRS. The SERRS effects of CAuNPs in both the dispersions and films suggest that their morphological factors are more important than the formation of junctions between particles to enhance the Raman scattering. Especially, the shapes of spectra from dispersions of the large CAuNPs resembled those from films (Figure S2). It is assumed that the large AuNPs couldn’t be packed well in the films, and then the junctions were not important in these systems. This is in consistent with the other reports which demonstrate the strong enhancing effects of non-spherical nanoparticles [34,35,36,37,42,43,46].

On the other hand, the Raman spectra from the film systems of spherical AuNPs were significantly enhanced than the dispersions, and a weak emission appeared in the range of 1500–1700 cm^−1^ (Figure 4C), different from the dispersion systems of spherical AuNPs (Figure 3C). These changes can be explained by the formation of hot-spots by accumulation of the spherical AuNPs in the film [26,27,28]. For the spherical AuNPs, the enhancing effect is dominantly due to the junctions formed between the particles. The smaller AuNPs could approach closer than the larger AuNPs and change their LSPR properties, as mentioned above for the changes in plasmon absorption bands before and after centrifugation (Figure 2). In details, the bands at 1508 cm^−1^ and 1645 cm^−1^ had similar intensities, which implies the intermediate between SERRS and non-resonance SERS [38,39].

It is worth noting that the bands at 1570 and 1600 cm^−1^ (vibration modes of phenyl group [39]) are almost equally enhanced on the films of spherical AuNPs, while the CAuNPs are less intensified the band at 1570 cm^−1^ than the other bands. From the viewpoint of the surface selection rule [27,28], this suggests that the twisting vibration mode of phenyl group in R6G (a Raman band at 1570 cm^−1^) is less resonated with the surface plasmon of CAuNPs than the vibration mode vertical to the xanthene ring (a Raman band at 1600 cm^−1^) [39]. That is, the R6G molecules adsorbed on CAuNPs are rather vertically oriented toward their surfaces [27,28], while the R6G molecules are randomly oriented in agglomerates of spherical AuNPs. For reference, the intensity relation of these bands was reverse on the SERRS of Ag nanoparticles, implying the different adsorption states of R6G between Au and Ag substrates [38,39,49].

In comparison with the SERRS in dispersion systems, this isotropic enhancing in agglomerates was also supported in the film systems of small CAuNPs: The bands in the range of 1400–1500 cm^−1^ were overlapped each other in the spectra from films (Figure S2). Thus, it can be assumed that the enhancing effect of small CAuNPs is more selective in the dispersions than on the films because of the less agglomeration. However, there was similar selectivity for the bands at 1570 and 1600 cm^−1^ both in dispersions and on films. Therefore, the SERRS bands in the range of 1400–1500 cm^−1^ were considered to be selectively enhanced by the interaction between R6G and AuNPs, since these bands could be for amine groups adsorbing on AuNPs [39]. The interaction should be sensitive to the adsorption states and the conformation of R6G molecule, and then the agglomeration in the film system caused the sift of SERRS bands of amine groups: For this interaction, the charge-transfer mechanism could be considered, although this mechanism is not yet clearly solved. Meanwhile, the SERRS bands of phenyl group was not sensitive to the environment, because the interaction of this moiety with AuNP should be indirect. Especially in the *J*-type aggregation of R6G, the molecules are oriented in the same direction, and then the sensitivity toward molecular conformation became significant [49].

### 3.4. Enhancement Factors of SERS in Dispersions and on Films

To evaluate the surface-enhancing effects, analytical enhancement factors (AEFs) were calculated for four major bands; the bands at 1183 cm^−1^ (C–H in-plane bending, xanthene ring and amine), 1363 cm^−1^ (C–C stretching, xanthene ring and amine), 1600 cm^−1^ (C–C stretching, phenyl ring), and 1645 cm^−1^ (C–C stretching, xanthene ring) [39]. The calculation of AEFs is based on the following formula [50]:

(1) $$AEF=\frac{I_{\mathrm{SERS}}}{I_{\mathrm{Raman}}}\times \frac{C_{\mathrm{Raman}}}{C_{\mathrm{SERS}}}$$

where *I*_SERS_: Intensity of enhanced band; *I*_Raman_: Intensity of corresponding band in the control solution without AuNPs; *C*_SERS_: Concentration of R6G in the mixture for the SERS measurement; *C*_Raman_: Concentration of R6G in the control solution without AuNPs.

**Figure 5:**
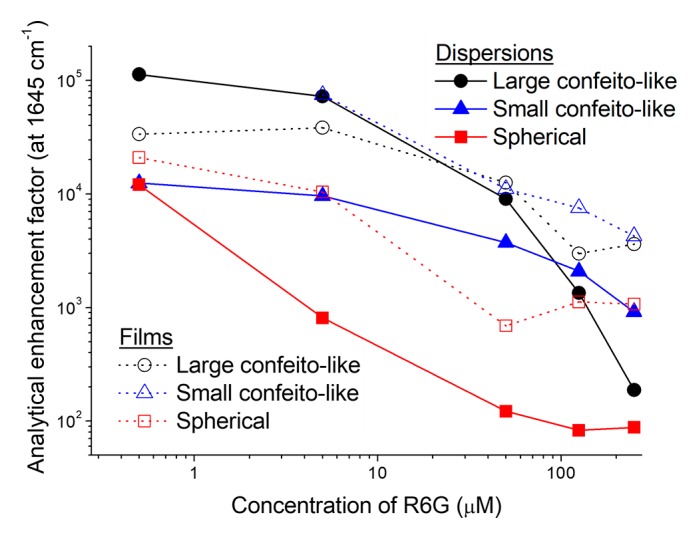
Analytical enhancement factors of Raman scattering (1645 cm^−1^) of R6G at various concentrations in the dispersions and on the films of AuNPs. The open-marks and dotted-lines represent the films, while closed-marks and solid-lines are for the dispersions.

The calculated AEFs were plotted against the concentration of R6G (Figure 5 and Figure S4).

The values of AEFs for the four bands had an order in common to all specimens (AuNPs in dispersions and on films) and various concentrations as below:

1183 cm^−^^1^ < 1363 cm^−^^1^ < 1600 cm^−^^1^ < 1645 cm^−^^1^ (2)

Generally, the SERS is strongly affected by the orientation of adsorbates on the surface and their interactions with surface plasmon [8]. Therefore, the similar behavior of these bands indicates that the orientation and interactions of R6G with AuNPs are basically the same to enhance these bands. The unique enhancement for the bands around 1400–1500 cm^−1^ could not be evaluated by the AEF, because these bands were not observed in the control.

**Figure 6:**
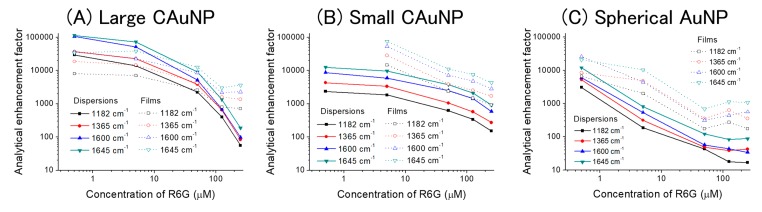
Analytical enhancing factors of Raman scattering of R6G at various concentrations in the dispersions and on the films of AuNPs. (**A**) large CAuNP, (**B**) small CAuNP, and (**C**) spherical AuNP. The open-marks and dotted-lines represent the films, while closed-marks and solid-lines are for the dispersions.

As the concentration of R6G increased, the AEFs consistently decreased in all bands of all specimens (Figure 6). The decrease of AEFs can usually be explained by the distance of target molecules from the surface of AuNP, because the molecules are excited by LSPR only in the vicinity of the AuNPs [7,8,27,28]. In the double logarithmic plot, the dispersions of CAuNPs (both of the large and the small ones) showed plateaus or slower decreases of AEFs than the dispersions of spherical AuNPs especially at low concentration (0.5–5 μM). Based on the Freundlich equation, this low concentration-dependency suggests that the CAuNPs adsorbs the R6G molecules more than the spherical AuNPs did [46]. For reference, spherical polymer nanoparticles (100 nm in diameter) can adsorb R6G with the molecular area of 1.6 nm^2^/dye; that is 20 × 10^3^ molecules of R6G can adsorb on the single nanoparticle [51]. From the density (19.3 g/cm^3^) and the atomic weight (197) of Au, the number of Au atoms in a spherical AuNP (100 nm in diameter) can be geometrically calculated as 30 million. Then, if this values are applied, the ratio of R6G/Au is 6.7 × 10^−4^. In this study, the dispersion contained 2.5 mM of Au, and then the large AuNP can adsorb ~1.6 μM of R6G to form the monolayer. Although the difference in the charge density and the surface morphology should be considered, the low concentration-dependency in this range seems to be reasonable.

On the other hand, the AEFs of spherical AuNPs changed to less than 1/10, as the concentration increased 10-fold from 0.5 μM to 5μM (Figure 5 and Figure 6). To the higher concentration (50 μM), this decline continued linearly in the double logarithmic plot, and it suggests that the hot-spots in the dispersion of spherical AuNPs are already saturated at 0.5 μM [46]. That is, the additional R6G could not contribute the SERS [7,8,27,28]. The hot-spots in the dispersion of spherical AuNPs should be the joints of nanoparticles randomly formed in the agglomerates [26,27,28], and their capacity is then limited in spite of the large specific surface area of spherical AuNPs.

The decreasing rates of AEFs in the dispersion of large CAuNPs was especially rapid against the increasing rate of concentration at the high concentration of R6G (from 50 μM to 250 μM) (Figure 5), and it suggests the precipitation of AuNPs, as mentioned above. For the small CAuNPs, this decrease of AEFs was slow over the change in concentration at high concentration (from 125 μM to 250 μM). Although the small CAuNPs also suffered the agglomeration as the R6G adsorbed, the small CAuNPs maintained the dispersibility at the higher concentration (~125 μM) than the large CAuNPs did (~50 μM). This approximately double concentration gap is consistency with the size-difference, that is, the specific surface area of the large (100 nm) and the small (50 nm) CAuNPs.

The large CAuNPs in dispersion had the strongest AEFs at the lowest concentration in the present study, which reached over 1 × 10^5^ for the band at 1645 cm^−1^ (Figure 5 and Figure 6). It is noteworthy that the AEFs in dispersion of large CAuNPs were stronger than those on the films at the low concentrations (0.5 and 5 μM). This could be explained by the blocking of hot-spots in the agglomerates on the films. On the other hand, the small CAuNPs had the maximum AEF of 1 × 10^4^ for 1645 cm^−1^ at 0.5 μM. In spite of their larger specific surface area, the small CAuNPs had rather weaker AEF than the large CAuNPs. This weakness can be attributed to the mismatching between the excitation wavelength (632.8 nm) and plasmon absorption bands (590 nm for isolated AuNPs and 800 nm for agglomerates (Figure 2) [31,40,41]. The agglomeration of AuNPs in the dispersion was expected to provide the hot-spots; however, the intensity of SERRS was not significantly increased due to this mismatching. Their AEFs were higher in the film systems than in the dispersion systems. This suggests that the advantages in film formation, e.g., concentration of AuNPs on the substrate, are superior to the negative effect of blocking. From this viewpoint, the isolated spherical AuNPs suffered the most mismatching between the excitation wavelength and the plasmon absorption band in this study, and the agglomeration in dispersions and the accumulation on the films could result in the shift of plasmon absorption band to the similar positon with that of the small CAuNPs. Although the characteristics of hot-spots in the films were not changed from that of dispersions, their number in the irradiated area could be increased by accumulation to intensify the AEFs for the films of spherical AuNPs (Figure 5).

Thus, the CAuNPs have advantages for ultrahighly sensitive analyses using SERRS: Their morphologies allow abundant adsorption of target molecules, and their plasmon absorption band can be tuned for the effective coupling with both the incident light and the emissions from adsorbate. Furthermore, the strong surface-enhancing effect in dispersions will be useful for applications in which film systems cannot be used.

## 4. Conclusions

SERS of R6G was measured in dispersions and on films of CAuNPs. The CAuNPs demonstrated strong SERS in both systems of dispersions and films, while the spherical Au nanoparticles had weak enhancing effects in both systems. The strong resonance between the excitation wavelength, surface plasmon, and molecular band allowed enhancing even the minor bands, which were not significantly enhanced by the spherical AuNPs. This difference suggested that the CAuNPs had hot-spots without agglomeration and enhanced the Raman scattering of R6G via the SERRS mechanism. The concentration-dependency of R6G on the SERS revealed that the CAuNPs had the stronger capture ability for SERS than the spherical AuNPs. The AEF of SERS depended on the size of the CAuNPs, and the larger particles (100 nm in diameter) showed stronger enhancing effect than the smaller particles (50 nm in diameter). The AEF reached to 1 × 10^5^ (at 1645 cm^−1^) for the dispersion of large CAuNPs, which was higher than that in film systems. Meanwhile, the small CAuNPs retained larger amount of adsorbate than the large CAuNPs did, due to the large specific surface area. These results suggest that the CAuNPs have advantages for ultrahighly sensitive devices, compared to the agglomerates of spherical AuNPs.

## Supplementary Materials

The following are available online at http://www.mdpi.com/1424-8220/17/11/2563/s1, Figure S1: Raman scattering of confeito-like AuNPs without R6G. Figure S2: Raman scattering of R6G at 50 μM in the dispersions and on the films of (A) small confeito-like AuNPs and (B) large confeito-like AuNPs. Figure S3: An optical image of SERS substrate (small confeito-like AuNPs and R6G at 50 µM).

## References

[1] Maier S.A., Atwater H.A. (2005). Plasmonics: Localization and guiding of electromagnetic energy in metal/dielectric structures. J. Appl. Phys..

[2] Jain P.K., Lee K.S., El-Sayed I.H., El-Sayed M.A. (2006). Calculated absorption and scattering properties of gold nanoparticles of different size, shape, and composition: Applications in biological imaging and biomedicine. J. Phys. Chem. B.

[3] Murray W.A., Barnes W.L. (2007). Plasmonic materials. Adv. Mater..

[4] Ho F.H., Wu Y.-H., Ujihara M., Imae T. (2012). A solution-based nano-plasmonic sensing technique by using gold nanorods. Analyst.

[5] Anker J.N., Hall W.P., Lyandres O., Shah N.C., Zhao J., Van Duyne R.P. (2008). Biosensing with plasmonic nanosensors. Nat. Mater..

[6] Eustis S., El-Sayed M.A. (2006). Why gold nanoparticles are more precious than pretty gold: Noble metal surface plasmon resonance and its enhancement of the radiative and nonradiative properties of nanocrystals of different shapes. Chem. Soc. Rev..

[7] Metiu H. (1984). Surface enhanced spectroscopy. Prog. Surf. Sci..

[8] Tian Z.Q., Ren B., Wu D.Y. (2002). Surface-enhanced Raman scattering: From noble to transition metals and from rough surfaces to ordered nanostructures. J. Phys. Chem. B.

[9] Zhang Z., Imae T., Sato H., Watanabe A., Ozaki Y. (2001). Surface-enhanced Raman scattering and surface-enhanced infrared absorption spectroscopic studies of a metalloporphyrin monolayer film formed on pyridine self-assembled monolayer-modified gold. Langmuir.

[10] Imae T., Zhang X. (2014). Effect of Au nanorod assemblies on surface-enhanced Raman spectroscopy. J. Taiwan Inst. Chem. Eng..

[11] Ataka K., Hara Y., Osawa M. (1999). A new approach to electrode kinetics and dynamics by potential modulated Fourier transform infrared spectroscopy. J. Electroanal. Chem..

[12] Imae T., Torii H. (2000). In Situ investigation of molecular adsorption on Au surface by surface-enhanced infrared absorption spectroscopy. J. Phys. Chem. B.

[13] Zhang Z., Imae T. (2001). Study of surface-enhanced infrared spectroscopy: 1. Dependence of the enhancement on thickness of metal island films and structure of chemisorbed molecules. J. Colloid Interface Sci..

[14] Zhang Z., Imae T. (2001). Study of surface-enhanced infrared spectroscopy: 2. Large enhancement achieved through metal–molecule–metal sandwich configurations. J. Colloid Interface Sci..

[15] Imae T., Takeshita T., Yahagi K. (2001). In Situ adsorption investigation of hexadecyltrimethylammonium chloride on self-assembled monolayers by surface plasmon resonance and surface enhanced infrared absorption spectroscopy. Stud. Surf. Sci. Catal..

[16] Zhang Z., Imae T. (2001). Surface enhanced infrared absorption and UV-vis spectroscopic study of a monolayer film of protoporphyrin IX zinc(II) on gold. Stud. Surf. Sci. Catal..

[17] Nagaoka H., Imae T. (2001). Poly(amido amine) dendrimer adsorption onto 3-mercaptopropionic acid self-assembled monolayer formed on Au surface -investigation by surface enhanced spectroscopy and surface plasmon sensing. Trans. Mater. Res. Soc. Jpn..

[18] Zhang Z., Imae T. (2001). Hydrogen-bonding stabilized self-assembled monolayer film of a functionalized diacid, protoporphyrin IX zinc(II), onto a gold surface. Nano Lett..

[19] Zhang Z., Yoshida N., Imae T., Xue Q., Bai M., Jiang J., Liu Z. (2001). A self-assembled monolayer of an alkanoic acid-derivatized porphyrin on gold surface: A structural investigation by surface plasmon resonance, ultraviolet–visible, and infrared spectroscopies. J. Colloid Interface Sci..

[20] Nagaoka H., Imae T. (2002). The construction of layered architectures of dendrimers—adsorption layers of amino-terminated dendrimers on 3-mercaptopropionic acid self-assembled monolayer formed on Au. Int. J. Nonlinear Sci. Numer. Simul..

[21] Ito M., Imae T., Aoi K., Tsutsumiuchi K., Noda H., Okada M. (2002). In Situ investigation of adlayer formation and adsorption kinetics of amphiphilic surface-block dendrimers on solid substrates. Langmuir.

[22] Ujihara M., Imae T. (2006). Adsorption behaviors of poly (amido amine) dendrimers with an azacrown core and long alkyl chain spacers on solid substrates. J. Colloid Interface Sci..

[23] Anger P., Bharadwaj P., Novotny L. (2006). Enhancement and quenching of single-molecule fluorescence. Phys. Rev. Lett..

[24] Mitamura K., Imae T., Tian S., Knoll W. (2008). Surface plasmon fluorescence investigation of energy-transfer-controllable organic thin films. Langmuir.

[25] Tam F., Goodrich G.P., Johnson B.R., Halas N.J. (2007). Plasmonic enhancement of molecular fluorescence. Nano Lett..

[26] Xu H., Aizpurua J., Kall M., Apell P. (2000). Electromagnetic contributions to single-molecule sensitivity in surface-enhanced Raman scattering. Phys. Rev. E.

[27] Moskovits M. (2005). Surface-enhanced Raman spectroscopy: A brief retrospective. J. Raman Spectrosc..

[28] Ghosh S.K., Pal T. (2007). Interparticle coupling effect on the surface plasmon resonance of gold nanoparticles: From theory to applications. Chem. Rev..

[29] Xu L., Kuang H., Xu C., Ma W., Wang L., Kotov N.A. (2012). Regiospecific plasmonic assemblies for In Situ raman spectroscopy in live cells. J. Am. Chem. Soc..

[30] Lu Y., Liu G.L., Kim J., Mejia Y.X., Lee L.P. (2005). Nanophotonic crescent moon structures with sharp edge for ultrasensitive biomolecular detection by local electromagnetic field enhancement effect. Nano Lett..

[31] Orendorff C.J., Gearheart L., Jana N.R., Murphy C.J. (2006). Aspect ratio dependence on surface enhanced Raman scattering using silver and gold nanorod substrates. Phys. Chem. Chem. Phys..

[32] Liu Z., Cheng L., Zhang L., Jing C., Shi X., Yang Z., Long Y., Fang J. (2014). Large-area fabrication of highly reproducible surface enhanced Raman substrate via a facile double sided tape-assisted transfer approach using hollow Au–Ag alloy nanourchins. Nanoscale.

[33] Kalachyova Y., Mares D., Jerabek V., Zaruba K., Ulbrich P., Lapcak L., Svorcik V., Lyutakov O. (2016). The effect of silver grating and nanoparticles grafting for LSP–SPP coupling and SERS response intensification. J. Phys. Chem. C.

[34] Kalachyova Y., Mares D., Jerabek V., Ulbrich P., Lapcak L., Svorcik V., Lyutakov O. (2017). Ultrasensitive and reproducible SERS platform of coupled Ag grating with multibranched Au nanoparticles. Phys. Chem. Chem. Phys..

[35] Hao E., Bailey R.C., Schatz G.C., Hupp J.T., Li S. (2004). Synthesis and optical properties of “Branched” gold nanocrystals. Nano Lett..

[36] Khoury C.G., Vo-Dinh T. (2008). Gold nanostars for surface-enhanced Raman scattering: Synthesis, characterization and optimization. J. Phys. Chem. C.

[37] Jeong G.H., Lee Y.W., Kim M., Han S.W. (2009). High-yield synthesis of multi-branched gold nanoparticles and their surface-enhanced Raman scattering properties. J. Colloid Interface Sci..

[38] Hildebrandt P., Stockburger M. (1984). Surface-enhanced resonance Raman spectroscopy of Rhodamine 6G adsorbed on colloidal silver. J. Phys. Chem..

[39] Watanabe H., Hayazawa N., Inouye Y., Kawata S. (2005). DFT vibrational calculations of rhodamine 6G adsorbed on silver: Analysis of tip-enhanced Raman spectroscopy. J. Phys. Chem. B.

[40] Itoh T., Yoshida K., Biju V., Kikkawa Y., Ishikawa M., Ozaki Y. (2007). Second enhancement in surface-enhanced resonance Raman scattering revealed by an analysis of anti-Stokes and Stokes Raman spectra. Phys. Rev. B.

[41] Yoshida K.I., Itoh T., Biju V., Ishikawa M., Ozaki Y. (2009). Experimental evaluation of the twofold electromagnetic enhancement theory of surface-enhanced resonance Raman scattering. Phys. Rev. B.

[42] Sharma J., Tai Y., Imae T. (2008). Biomodulation approach for gold nanoparticles: Synthesis of anisotropic to luminescent particles. J. Phys. Chem. C.

[43] Ujihara M., Imae T. (2013). Versatile one-pot synthesis of confeito-like Au nanoparticles and their surface-enhanced Raman scattering effect. Colloids Surf. A.

[44] Ujihara M., Dang N.M., Imae T. (2014). Fluorescence quenching of uranine on confeito-like Au nanoparticles. J. Nanosci. Nanotechnol..

[45] Ujihara M., Dang N.M., Chang C.C., Imae T. (2014). Surface-enhanced infrared absorption spectra of eicosanoic acid on confeito-like Au nanoparticle. J. Taiwan Inst. Chem. Eng..

[46] Chang C.C., Imae T., Chen L.Y., Ujihara M. (2015). Efficient surface enhanced Raman scattering on confeito-like gold nanoparticle-adsorbed self-assembled monolayers. Phys. Chem. Chem. Phys..

[47] Kimling J., Maier M., Okenve B., Kotaidis V., Ballot H., Plech A. (2006). Turkevich method for gold nanoparticle synthesis revisited. J. Phys. Chem. B.

[48] Salleres S., Arbeloa F.L., Martínez V.M., Arbeloa T., Arbeloa Í.L. (2009). Improving the fluorescence polarization method to evaluate the orientation of fluorescent systems adsorbed in ordered layered materials. J. Lumin..

[49] Zhao J., Jensen L., Sung J., Zou S., Schatz G.C., Van Duyne R.P. (2007). Interaction of plasmon and molecular resonances for rhodamine 6G adsorbed on silver nanoparticles. J. Am. Chem. Soc..

[50] Le Ru E.C., Blackie E., Meyer M., Etchegoin P.G. (2007). Surface enhanced raman scattering enhancement factors: A comprehensive study. J. Phys. Chem. C.

[51] Charreyre M.-T., Zhang P., Winnik M.A., Pichot C., Graillat C. (1995). Adsorption of Rhodamine 6G onto polystyrene latex particles with sulfate groups at the surface. J. Colloid Interface Sci..

