# Supplementary material for: Surface-Enhanced Resonance Raman Scattering of Rhodamine 6G in Dispersions and on Films of Confeito-Like Au Nanoparticles
Source: Sensors (Basel). 2017 Nov 7;17(11):2563. doi: 10.3390/s17112563 (PMC5712894; doi:10.3390/s17112563)
Supplement: Supplementary file 1 [file sensors-17-02563-s001.pdf]

Supporting Information

Surface-Enhanced Resonance Raman Scattering of Rhodamine 6G in Dispersions and on Films of Confeito-Like Au Nanoparticles

Masaki Ujihara[1,*], Nhut Minh Dang [2] and Toyoko Imae [1,2]

[1] Graduate Institute of Applied Science and Technology, National Taiwan University of Science and Technology, 43 Keelung Road, Section 4, Taipei 10607, Taiwan, and

[2] Department of Chemical Engineering, National Taiwan University of Science and Technology, 43 Keelung Road, Section 4, Taipei 10607, Taiwan

Corresponding author: masaki.ujihara@mail.ntust.edu.tw

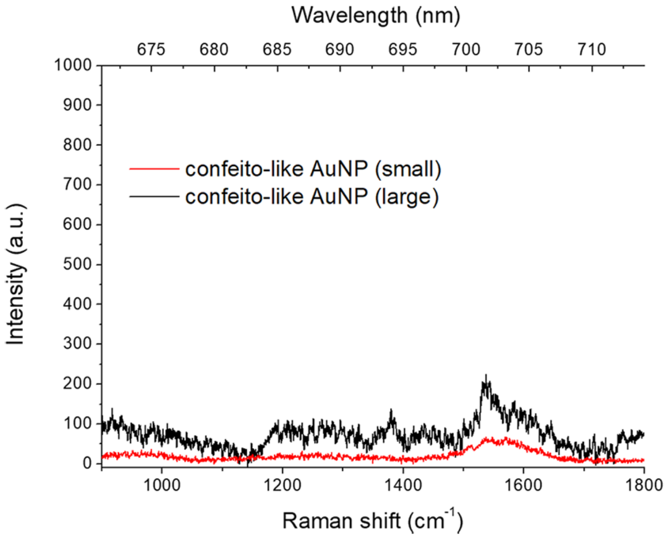

Figure S1. Raman scattering of confeito-like AuNPs without R6G.

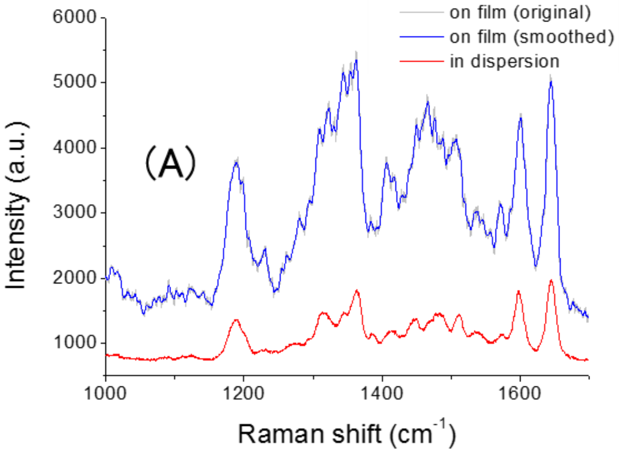

Figure S2. *Cont.*

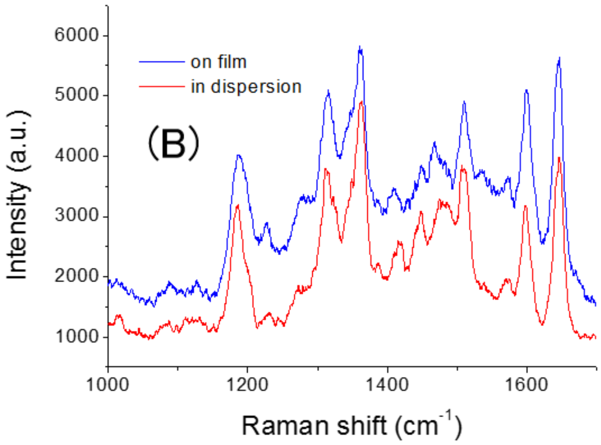

Figure S2. Raman scattering of R6G at 50 μM in the dispersions and on the films of (A) small confeito-like AuNPs and (B) large confeito-like AuNPs. In (A), original spectrum (gray) was smoothed by the adjacent average method with 10 points (corresponding to ±1.7 cm^{-1}).

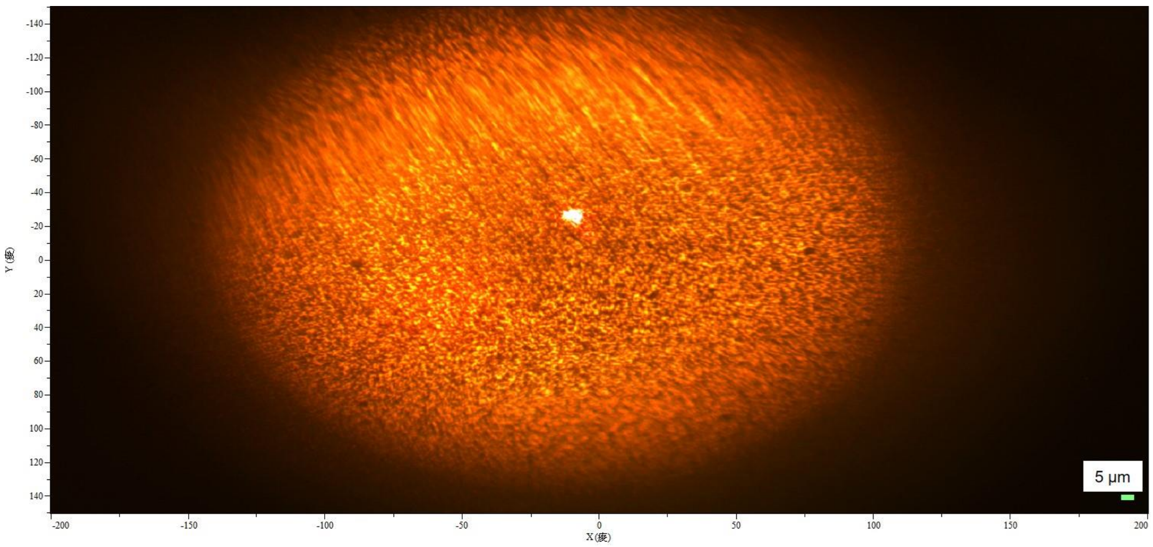

Figure S3. An optical image of SERS substrate (small confeito-like AuNPs and R6G at 50 μM). A white spot in the center was the laser spot for focusing. The confeito-like AuNPs were observed as flocks.
